# Effects of Glutamate and Aspartate on Serum Antioxidative Enzyme, Sex Hormones, and Genital Inflammation in Boars Challenged with Hydrogen Peroxide

**DOI:** 10.1155/2016/4394695

**Published:** 2016-09-29

**Authors:** Hengjia Ni, Lu Lu, Jinpin Deng, Wenjun Fan, Tiejun Li, Jiming Yao

**Affiliations:** ^1^Key Laboratory for Agro-Ecological Processes in Subtropical Region, Hunan Research Center of Livestock and Poultry Sciences, South Central Experimental Station of Animal Nutrition and Feed Science in the Ministry of Agriculture, Institute of Subtropical Agriculture, The Chinese Academy of Sciences, Hunan, China; ^2^Department of Animal Science, Hunan Agriculture University, Hunan, China; ^3^Department of Animal Science, South China Agriculture University, Guangdong, China; ^4^Guangdong Wangda Group Co., Ltd., Guangdong, China; ^5^Guangdong Wangda Group Academician Workstation for Clean Feed Technology Research and Development in Swine, Guangdong, China; ^6^Hunan Co-Innovation Center of Animal Production Safety, Hunan, China

## Abstract

*Background*. Oxidative stress is associated with infertility. This study was conducted to determine the effects of glutamate and aspartate on serum antioxidative enzymes, sex hormones, and genital inflammation in boars suffering from oxidative stress.* Methods*. Boars were randomly divided into 4 groups: the nonchallenged control (CON) and H_2_O_2_-challenged control (BD) groups were fed a basal diet supplemented with 2% alanine; the other two groups were fed the basal diet supplemented with 2% glutamate (GLU) or 2% aspartate (ASP). The BD, GLU, and ASP groups were injected with hydrogen peroxide (H_2_O_2_) on day 15. The CON group was injected with 0.9% sodium chloride solution on the same day.* Results*. Dietary aspartate decreased the malondialdehyde (MDA) level in serum (*P* < 0.05) compared with the BD group. Additionally, aspartate maintained serum luteinizing hormone (LH) at a relatively stable level. Moreover, glutamate and aspartate increased transforming growth factor-*β*1 (TGF-*β*1) and interleukin-10 (IL-10) levels in the epididymis and testis (*P* < 0.05) compared with the BD group.* Conclusion*. Both glutamate and aspartate promoted genital mRNA expressions of anti-inflammatory factors after oxidative stress. Aspartate more effectively decreased serum MDA and prevented fluctuations in serum sex hormones after H_2_O_2_ challenge than did glutamate.

## 1. Introduction

Artificial insemination has been shown to be a successful reproductive management approach to improve livestock production efficiency. Artificial insemination facilitates the use of high-genetic-merit boars for inseminating a group of sows [1]. Therefore, boars with a high reproductive capacity can improve efficiency with significant genetic effects [2].

However, many risk factors can cause reproductive dysfunction in boars, such as harsh environments and mental stress and disease [3–6]. Cumulative evidence indicates that whole body oxidative stress (OS) is related to all these risk factors. The imbalance between oxidation and antioxidation and the elevation of reactive oxygen species (ROS) are believed to cause defective spermatogenesis and sperm dysfunction in sexually mature boars [7]. Approximately 25% of infertile men showed high levels of semen ROS, while fertile men did not [8]. In mammals, spermatozoal membranes have many polyunsaturated fatty acids (PUFAs) and are sensitive to ROS attack, which can damage membrane and morphological integrity, impair cellular functions, and promote sperm apoptosis and impaired sperm motility [7]. Additionally, most infertile men have been shown to suffer from acute or chronic inflammation of the genitourinary tract [9]. Cytokines and ROS may interact in mediating the toxic effects of inflammation [10]. Previous reports found a positive correlation between seminal ROS generation and seminal plasma proinflammatory cytokines, such as interleukin-8 (IL-8), interleukin-6 (IL-6), and tumor necrosis factor-*α* (TNF-*α*) [11]. A sustained inflammatory/oxidative environment can damage healthy cells, which may lead to infertility and cause economic losses. In light of these factors, a strategy to minimize oxidative stress and genital inflammation in boars is required.

In recent years, many antioxidant therapies have been used to enhance reproductive ability [12–14]. These compounds can directly and indirectly influence the concentration of ROS metabolism-regulating processes [15–17]. As nutritional supplements for animals, glutamate and aspartate were found to possess antioxidative activity. Dietary supplementation with 2% glutamate enhanced the antioxidant system and improved body weight in piglets after diquat challenge [18], suggesting it can ameliorate the damage caused by acute oxidative stress. Moreover, glutamate modulated the body weight [19], regulated the release of hormones [20] and lipid metabolism [21], and improved gastrointestinal absorption [22]. Dietary supplementation with aspartate at a dose of 0.5–1% restored the intestinal barrier, improved liver metabolism, and enhanced energy status in piglets challenged with lipopolysaccharide [23, 24]. These effects may be due to its contributions to the tricarboxylic acid cycle and the production of ATP [25]. Because glutamate and aspartate have beneficial health and antioxidative effects, they may protect boars suffering from acute oxidative stress. Thus, we evaluated the effects of glutamate and aspartate supplementation on the antioxidative enzymes and reproductive system of boars under conditions of acute oxidative stress. Hydrogen peroxide (H_2_O_2_) was introduced to establish the oxidative stress model [26].

## 2. Materials and Methods

### 2.1. Experimental Design

Sixteen large white boars (6 months old, 85 ± 3.2 kg BW) were housed in individual metabolic cages equipped with a nipple drinker and a feeder in the room, and the temperature was maintained at 25°C. All boars were fed a basal diet (Table 1), which was formulated to meet the nutritional needs of 6-month-old boars according to NRC (1998), for 5 days. Then, they were divided into 4 groups to undergo different treatments: (1) nonchallenged control (CON, basal diet + 2% alanine, saline-challenged); (2) H_2_O_2_-challenged control (BD, basal diet + 2% alanine, H_2_O_2_-challenged); (3) H_2_O_2_ + 2% glutamate treatment (GLU, basal diet + 2% glutamate, H_2_O_2_-challenged); and (4) H_2_O_2_ + 2% aspartate treatment (ASP, basal diet + 2% aspartate, H_2_O_2_-challenged). Three diets were prepared to be isonitrogenous by introducing alanine into the basal diet. All groups were treated with a single intraperitoneal injection (i.p.) of 10% H_2_O_2_ (1 mL/kg body weight) on day 15, except for the CON group, which was injected with the same volume of 0.9% sodium chloride solution. The dosage of H_2_O_2_ used in this study was based on a previous study [26]. The amino acids (purity > 99%) used in this study were purchased from Beijing Chemclin Biotech Co., Ltd. (Beijing, China). Other chemicals were purchased from Sinopharm Chemical Reagent Co., Ltd. (Beijing, China).

### 2.2. Sample Collection

All boars were anesthetized using sodium pentobarbital and then killed by jugular puncture on day 22. The blood, testis, and epididymis samples were collected. This study was performed in accordance with the Declaration of Helsinki and ratified by the Laboratory Animal Care Advisory Committee at the Institute of Subtropical Agriculture, the Chinese Academy of Sciences [31]. Blood was collected from the jugular vein of boars. Serum samples were prepared by centrifugation of blood samples at 2000 rpm (or 500 ×g) for 10 min at 4°C and then stored at −80°C until use. The testis and epididymis were weighed after slaughter, and the organ coefficient was calculated as follows [32]: organ coefficient (%) = organ weight/body weight of boar × 100%. A small portion of the testis and epididymis was immediately frozen in liquid nitrogen and maintained at −80°C for subsequent analyses of gene expression.

### 2.3. Measurements of Specific Enzymes and Hormones in Serum

The serum concentrations of superoxide dismutase (SOD), glutathione peroxidase (GSH-Px), and malondialdehyde (MDA) were analyzed to determine the serum antioxidant capacity. They were measured using kits from the Nanjing Jiancheng Bioengineering Institute (Nanjing, China) [33]. Kits from Beijing North Institute of Biological Technology (Beijing, China) were used to determine the serum concentrations of reproductive hormones, such as follicle-stimulating hormone (FSH), luteinizing hormone (LH), and testosterone (T2) [34].

### 2.4. RNA Extraction and cDNA Synthesis

Total mRNA from liquid nitrogen-pulverized testis and epididymis was extracted using TRIzol reagent (Invitrogen, USA) according to the manufacturer's recommendation [35]. RNA integrity was confirmed by agarose gel electrophoresis. The RNA concentrations were determined by measuring the absorbance at 260 nm in a spectrophotometer [36]. Reverse transcription was performed with a 2 mg RNA sample using PrimeScript*™* RT reagent kit (TaKaRa) according to the manufacturer's instructions. The cDNA was synthesized with a PrimeScript 1st-Strand cDNA Synthesis Kit (TaKaRa, Japan) [37].

### 2.5. Quantification of mRNA by Real-Time PCR Analysis

Primers were designed with Primer 5.0 based on the cDNA sequence of boars to amplify target DNA (Table 2). GADPH was used as a reference gene to normalize target gene transcript levels. Real-time PCR was conducted with a total volume of 25 *μ*L containing 12.5 *μ*L SYBR® Premix Ex Taq (Tli RNase H* Plus*), 2 *μ*L template (<100 ng), and 1 *μ*L of each of the forward and reverse primers (10 *μ*M). The PCR protocol was 10 min at 95°C, followed by 40 cycles of 95°C for 15 s, 60°C for 30 s, and 72°C for 30 s. Amplification efficiency for each target gene was determined by plotting the threshold cycle (Ct) versus log (initial cDNA). The relative quantification of target gene expression was evaluated by 2^−ΔΔCT^ method [36].

### 2.6. Statistical Analysis

All statistical analyses were carried out using SigmaPlot 12 software. First, tests for normal distribution (Shapiro-Wilk test) and equal variance were performed. If both tests were positive, one-way analysis of variance was used followed by post hoc Tukey's test. Values with different letter are significantly different (*P* < 0.05), while values with the same letter are not significantly different (*P* > 0.05).

## 3. Results

### 3.1. Organ Coefficient

The final body weights and testis coefficients are shown in Table 3. The H_2_O_2_ challenge did not affect the body weight of boars but increased the testis coefficient 7 days after the treatment compared with the CON group (*P* < 0.05). However, diets with and without glutamate and aspartate supplementation had no significant impact on the testis coefficient (*P* > 0.05).

### 3.2. Concentrations of MDA, SOD, and GSH-Px in Serum

**Figure 1:**
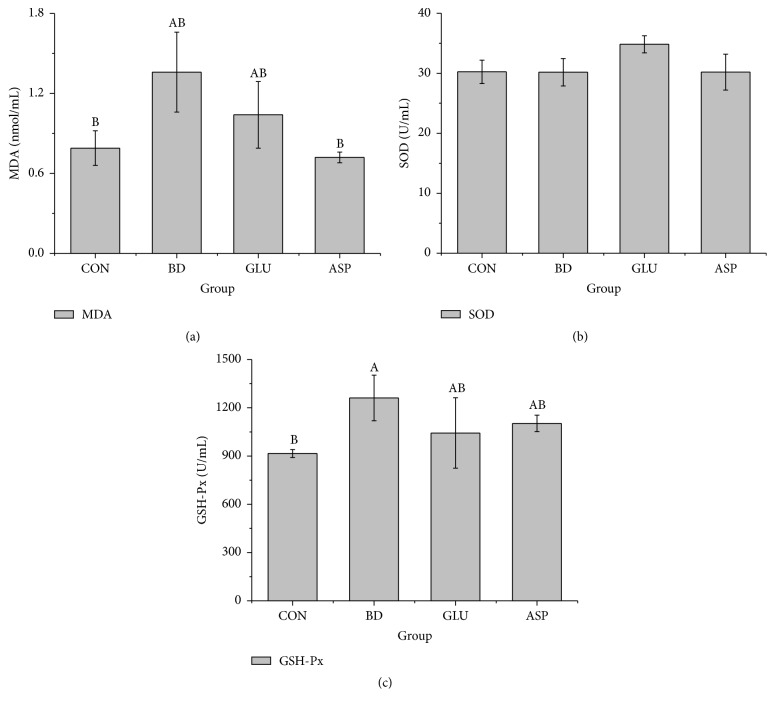
The effects of glutamate and aspartate on serums SOD, MDA, and GSH-Px after H_2_O_2 _challenge. SOD: superoxide dismutase; MDA: malondialdehyde; and GSH-Px: glutathione peroxidase. Values are means (*n* = 4), with their standard deviation represented by vertical bars. ^A, B, C^Mean values with different letters were significantly different (*P* < 0.05).

Figure 1 shows that the H_2_O_2_ administration disturbed the balance between oxidation and antioxidation in boars. The MDA concentration in serum significantly increased after H_2_O_2_ challenge compared with the CON group (*P* < 0.05). Dietary aspartate significantly reduced the MDA level in serum compared with the BD group (*P* < 0.05), and the value was similar to that of the CON group (*P* > 0.05). Dietary glutamate slightly decreased the MDA level in serum, but the changes were not significant compared with the BD group (*P* > 0.05).

Intraperitoneal injection with H_2_O_2_ did not affect the SOD level in serum, and dietary supplementation with glutamate and aspartate also had little influence on SOD concentration (*P* > 0.05). The serum GSH-Px concentration in the BD group was significantly increased 7 days after H_2_O_2_ challenge compared with the CON group (*P* < 0.05). Dietary supplementation with glutamate and aspartate had little effect on the GSH-Px level compared with the BD group (*P* > 0.05).

### 3.3. Sex Hormones in Serum

**Figure 2:**
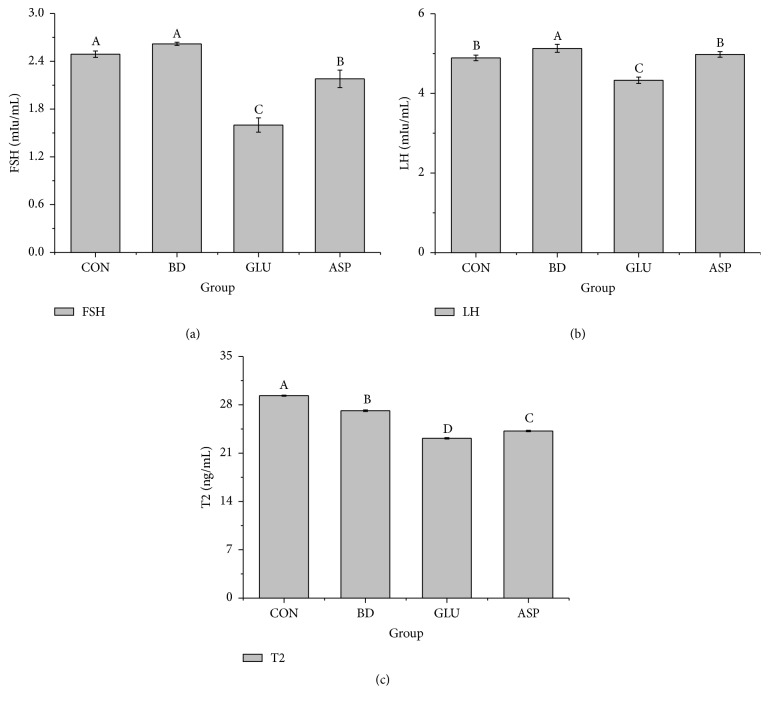
The effects of glutamate and aspartate on serum sex hormones (FSH, LH, and T2) after H_2_O_2_ challenge. FSH: follicle-stimulating hormone; T2: testosterone; and LH: luteinizing hormone. Values are means (*n* = 4), with their standard deviation represented by vertical bars. ^A, B, C, D^Mean values with different letters were significantly different (*P* < 0.05).

The concentrations of FSH, LH, and T2 in boar serum were determined, and the results are shown in Figure 2. The H_2_O_2_ challenge had little impact on serum FSH, but supplementation with glutamate and aspartate significantly decreased the FSH level in serum compared with the CON group (*P* < 0.05). In contrast to FSH, the LH concentration was significantly higher, and the T2 level was significantly lower in the BD group than that in the CON group (*P* < 0.05). Dietary aspartate maintained serum LH at a stable level under oxidative stress, and its value showed no significant difference from that of the CON group (*P* > 0.05). However, dietary supplementation with glutamate and aspartate significantly decreased the serum T2 level compared with the BD group (*P* < 0.05).

### 3.4. Expression of Inflammatory Genes in Testis and Epididymis

**Figure 3:**
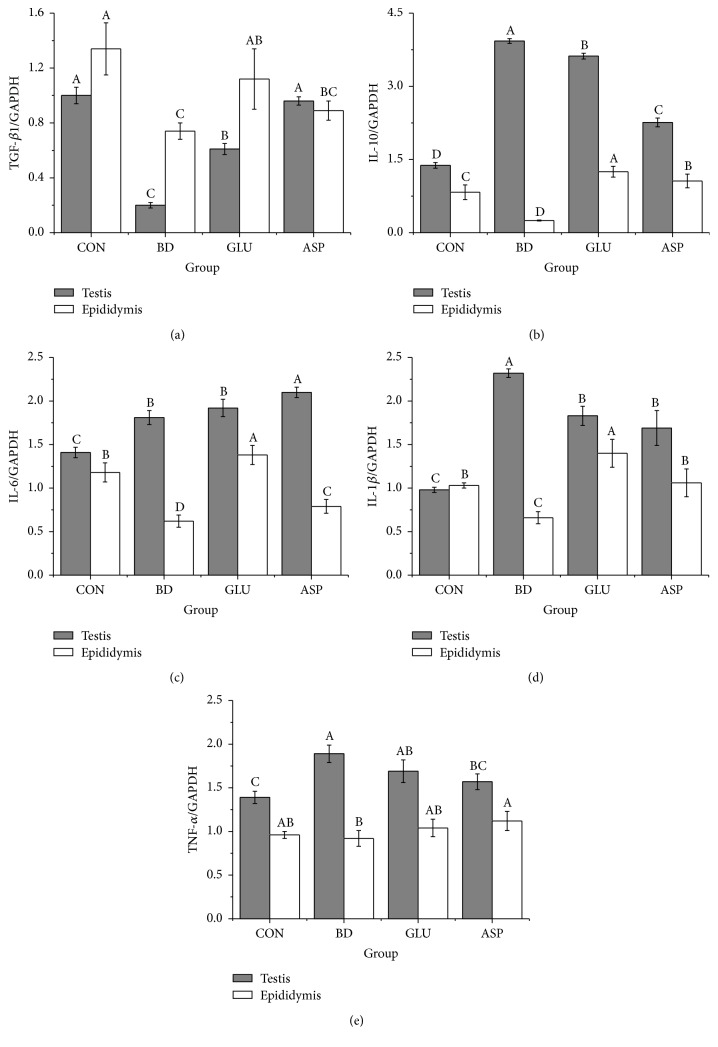
The relative mRNA expression of inflammatory-related factors (TGF-*β*1, IL-10, IL-6, IL-1*β*, and TNF-*α*) in boar's testis and epididymis after H_2_O_2_ challenge. TGF-*β*1: transforming growth factor-*β*1; TNF-*α*: tumor necrosis factor-*α*; and IL: interleukin. Values are means (*n* = 4), with their standard deviation represented by vertical bars. ^A, B, C, D^Mean values with different letters were significantly different (*P* < 0.05).

The relative mRNA expressions of inflammatory factors (TGF-*β*1, IL-10, IL-6, IL-1*β*, and TNF-*α*) in boar testis and epididymis after H_2_O_2_ challenge were analyzed. The results (Figure 3) showed that inflammatory factors (IL-6, IL-1*β*, and TNF-*α*) were markedly upregulated in the testis after H_2_O_2_ challenge compared with the CON group. Dietary glutamate failed to decrease the IL-6 and TNF-*α* expressions in the testis and epididymis compared with the BD group, but it significantly upregulated TGF-*β*1 in the testis and both TGF-*β*1 and IL-10 in epididymis compared with the BD group (*P* < 0.05). Dietary aspartate upregulated TGF-*β*1 expression in the testis and IL-10 expression in the epididymis compared with the BD group (*P* < 0.05). Additionally, dietary aspartate decreased the IL-1*β* and TNF-*α* expressions in testis compared with the BD group (*P* < 0.05).

## 4. Discussion

ROS are products of normal cellular metabolism. However, once the balance between the generation of ROS and antioxidant scavenging activity is disturbed, oxidative stress occurs [38, 39]. Many studies have indicated that the most common ROS, such as H_2_O_2_, ROO^−^, and OH^−^, can lead to sperm damage and deformity and eventually male infertility [7]. Lipids are considered the most susceptible biomolecules and are abundant in the sperm plasma membrane and other cell membranes in the form of polyunsaturated fatty acids (PUFAs) [40]. ROS attack these PUFAs, leading to lipid peroxidation and elevated generation of MDA, which has been used to monitor the degree of peroxidative damage [40]. H_2_O_2_ is a highly reactive oxygen species. It can freely disperse into the mitochondria and lead to the generation of massive ROS levels. Peritoneal administration of 10% H_2_O_2_ (1 mL/kg body weight) resulted in oxidative stress [26]. Consistent with a previous study, the data from Figure 1 show that H_2_O_2_ injection (BD group) increased the serum MDA concentration compared with the CON group, suggesting that H_2_O_2_ had successfully induced systemic oxidative stress in boars. Notably, dietary glutamate and aspartate were reported to significantly alleviate the oxidative stress of piglets seven days after H_2_O_2_ or diquat challenge [18, 26] and would possibly have a better effect with prolonged use. Thus, to protect against oxidative stress, we fed the boars 2% glutamate or 2% aspartate prior to the H_2_O_2_ challenge. All boars were slaughtered and sampled seven days after H_2_O_2_ challenge to determine whether glutamate and aspartate rapidly alleviated the oxidative stress in boars as they did in piglets [26].

To study the effects of glutamate and aspartate on boars under oxidative stress, we analyzed their impact on serum sex hormones, serum antioxidative enzymes, and genital inflammation. Hormones are signaling molecules. They can be transported to distant organs to regulate physiology and behavior via the circulatory system. The biosynthesis and secretion of hormones are regulated by other hormones, plasma concentrations of ions or nutrients, neurons and mental activity, and environmental changes [41]. Changes in hormone concentrations can also reflect the state of homeostasis [42]. Testosterone (T2) is a male sex hormone and is predominantly produced in the testis. It has an important role in sexual and reproductive development [43]. Fluctuations in T2 levels affect the sex drive, sperm production, and fat distribution [43, 44] and are associated with overall health in boars. A previous study revealed that testicular inflammation was related to a significant decrease in T2 production [45]. In this study, the H_2_O_2_ challenge decreased the serum T2 level in the BD group compared with that in the CON group, and administration of glutamate and aspartate failed to increase the T2 level. Combining the results of inflammatory gene analysis in testis, we found similar results indicating that H_2_O_2_ caused testicular inflammation along with downregulated T2 levels in serum. However, dietary glutamate and aspartate had little effect on testicular inflammation.

The course of normal spermatogenesis not only relies on the testicular secretion of T2 but also relies on the normal pituitary secretion of FSH and LH [46]. In males, FSH stimulates a number of downstream targets in Sertoli cells to affect spermatogenesis. Conversely, Sertoli cells are stimulated by FSH, which produces inhibin. This compound provides negative feedback to the anterior pituitary to decrease FSH secretion. LH stimulates Leydig cells to produce T2, which provides negative feedback to the anterior pituitary and hypothalamus [47]. In this study, a slight fluctuation in LH level was observed in the serum after H_2_O_2_ injection. However, it is difficult to determine the physiological significance of changes in these hormones because the mechanisms of FSH and LH regulation in boars under oxidative stress are still unclear. However, we analyzed the effect of glutamate or aspartate on preventing hormone disorders under oxidative stress by comparing the effects of different treatments on boar hormone concentrations. The results suggested that aspartate had positive effect on maintaining serum LH at a relatively stable level.

Glutamate administration can alleviate diquat-induced oxidative stress by enhancing SOD and T-AOC levels and inhibiting lipid oxidation and MDA generation [18]. Nevertheless, several studies suggested that glutamate accumulation in the brain increases oxidative stress [48]. Glutamate accumulation also has been reported to generate NO and stimulate cyclic guanosine monophosphate formation. Therefore, the relationship between glutamate and ROS appears to be complex [48]. Addition of aspartate was reported to prevent growth suppression of weaned pigs after LPS challenge [24]. However, our previous study found that administration of aspartate did not facilitate growth performance and showed little effect on relieving oxidative stress induced by diquat [18]. In this study, we found that aspartate was capable of reducing the MDA level in boar serum, while glutamate failed to alleviate H_2_O_2_-induced oxidative stress in boars. Boars in the BD group even had higher serum GSH-Px level than those in the GLU group. Whether these differences in their effects on oxidative stress are due to the distinct gender and age of the pigs requires further investigation.

Inflammatory factors are involved in oxidative stress [49]. Previous studies have shown that inflammation is a manifestation of increased oxidative stress [49]. Conversely, inflammatory cells also produce many mediators, such as metabolites of arachidonic acid, chemokines, and cytokines, which further recruit inflammatory cells to the site of injury and produce more ROS [49]. Under these circumstances, antioxidants in seminal plasma help prevent oxidative stress [50]. However, seminal plasma antioxidants cannot reach the testis, and the sperm must rely on epididymal/testicular antioxidants and their own intrinsic antioxidant capacity for protection during spermatogenesis and epididymal storage [51]. Glutamate and aspartate belong to the arginine family of amino acids, as well as proline, glutamine, asparagine, ornithine, citrulline, and arginine. They are interconvertible via complex interorgan metabolism in most mammals, including pigs. Both of these amino acids are predominantly absorbed in the small intestine [52]. However, the intestinal mucosa will preferentially use dietary glutamate rather than other amino acids [53]. Once glutamate and aspartate are absorbed by enterocytes, they are utilized as fuels or participate in the synthesis of other amino acids, such as alanine, arginine, and others, and then enter the systemic circulation [26]. Notably, arginine affects purine metabolism in testis tissues by activation of adenosine production, the salvage pathway, and ATP regeneration and shows protective effects on male metabolic and reproductive function [54]. Thus, dietary glutamate and aspartate are believed to be beneficial in enhancing testicular defense systems and reducing epididymal/testicular inflammation induced by oxidative stress.

To evaluate the effect of dietary glutamate and aspartate on genital inflammation in boars, we determined the mRNA expressions of TGF-*β*1, IL-10, IL-6, IL-1*β*, and TNF-*α* in boar testis and epididymis samples. TGF-*β*1 is a key regulator of male reproductive function [55]. The steroidogenesis of Leydig cells, the organization of peritubular myoid cells, and testis development and spermatogenesis are all modulated by testicular TGF-*β*1, and it is also involved in the tight balance between proliferative and apoptotic responses in the Leydig cells [55]. Another anti-inflammatory cytokine, IL-10, plays a role in upregulating monocyte production of soluble TNF-*α* and the IL-1*β* receptor antagonist [56]. It protects endothelial function after an acute inflammatory stimulus by limiting local increases in superoxide anion [57]. In this study, dietary supplementation with glutamate and aspartate caused varying degrees of upregulation of TGF-*β*1 and IL-10 mRNA expression in the testis and epididymis. These findings suggested that glutamate and aspartate protect boar testis and epididymis from inflammation by increasing TGF-*β*1 and IL-10 levels. However, the organ coefficient results also showed that there was a slight swelling in boar testis after H_2_O_2_ challenge, suggesting that the testes were suffering from chronic inflammation. Even when boars were fed glutamate or aspartate, the swelling was not being relieved. The testicular inflammation indicated that the production and release of large amounts of ROS can trigger the immune responses and stimulate the secretion of numerous biological substances (such as leukocytes), which resulted in increased inflammation [9]. Because the antioxidant capacity of the testis and epididymis is very important in preventing oxidative stress-induced damage [7], the amount and activity of major antioxidant enzymes, especially SOD, catalase, and glutathione/glutathione peroxidase (GSH-Px), in the testis and epididymis are of great importance. Whether the testicular inflammation we found in this study was caused by the failure to elevate antioxidant enzyme activities in testis requires further research.

In conclusion, this study showed that dietary supplementation with glutamate and aspartate had little effect on increasing SOD and GSH-Px concentrations. They also failed to maintain FSH and T2 at a stable level in serum. Both glutamate and aspartate were unable to decrease the mRNA expressions of inflammatory factors (IL-1*β*, IL-10, and TNF-*α*) in testis and epididymis after H_2_O_2_ challenge. However, glutamate and aspartate promoted the genital mRNA expressions of anti-inflammatory factors (TGF-*β*1 and IL-10) after oxidative stress. Aspartate was more effective than glutamate in decreasing MDA levels and preventing the fluctuations of LH in boar serum.

## Figures and Tables

**Table 1:** The composition of basal diet.

Items	Basal diet
Ingredient (%)	
Corn	64
Soybean meal	22
Wheat bran	6
Fish meal	4
Premix^*∗*^	4

Composition	
Crude protein (%)	13.80
Metabolism energy (MJ/kg)	13.25
Calcium (%)	0.85
Phosphorus (%)	0.72

^*∗*^Composition: vitamin A, 400000 U; vitamin D, 380000 U; vitamin E, 1200 U; vitamin K, 360 mg/kg; vitamin B_1_, 145 mg/kg; vitamin B_2_, 135 mg/kg; vitamin B_6_, 85 mg/kg; vitamin B_12_, 0.58 mg/kg; niacin, 600 mg/kg; calcium pantothenate, 350 mg/kg; folate, 90 mg/kg; biotin, 12 mg/kg; choline chloride, 15 g; copper, 0.4 g; iron, 3.3 g; manganese, 0.5 g; cobalt, 10 mg; iodine, 10 mg; and selenium, 8 mg.

**Table 2:** Primers used in this study.

Gene^1^	Accession number	Primer squence (5′-3′)	Size (bp)
IL-6	NM_001252429.1	F: CCTCTCCGGACAAAACTGAA R: TCTGCCAGTACCTCCTTGCT	118 [27]

IL-10	NM_214041.1	F: CTGCCTCCCACTTTCTCTTG R: TCAAAGGGGCTCCCTAGTTT	95 [28]

IL-1*β*	NM_214055.1	F: AGTGGAGAAGCCGATGAAGA R: CATTGCACGTTTCAAGGATG	113

TGF-*β*1	NM_214015.1	F: TTT CGC CTC AGT GCC CA R: GCCAGAATTGAACCCGTTAA	78 [27]

TNF-*α*	NM_214022.1	F: CCACGCTCTTCTGCCTACTGC R: GCTGTCCCTCGGCTTTGAC	168 [29]

GAPDH	NM_001206359.1	F: AAGGAGTAAGAGCCCCTGGA R: TCTGGGATGGAAACTGGAA	140 [30]

^1^IL-6: interleukin-6; IL-10: interleukin-10; IL-1*β*: interleukin-1*β*; TGF-*β*1: transforming growth factor-*β*1; TNF-*α*: tumor necrosis factor-*α*; GAPDH: glyceraldehyde-3-phosphate dehydrogenase.

**Table 3:** Final body weight, testis weight, and testis coefficient after H_2_O_2_ challenge.

	CON	BD	GLU	ASP
Final BW (kg)	82.67 ± 2.52	87.67 ± 1.53	87.67 ± 6.35	81.00 ± 2.00
TW (g)	137.07 ± 15.86^b^	343.23 ± 38.06^a^	375.40 ± 60.51^a^	321.13 ± 70.67^a^
T coefficient (%)	0.17 ± 0.02^b^	0.39 ± 0.05^a^	0.43 ± 0.04^a^	0.40 ± 0.09^a^

BW = body weight; TW = testis weight; and T coefficient = testis coefficients.

Values are means (*n* = 4), with their standard deviation represented by mean ± STD. ^a,b^Mean values with different letters were significantly different (*P* < 0.05).

## References

[1] Maes D., Nauwynck H., Rijsselaere T. (2008). Diseases in swine transmitted by artificial insemination: an overview. *Theriogenology*.

[2] Didion B. A., Kasperson K. M., Wixon R. L., Evenson D. P. (2009). Boar fertility and sperm chromatin structure status: a retrospective report. *Journal of Andrology*.

[3] Smital J. (2009). Effects influencing boar semen. *Animal Reproduction Science*.

[4] Kim B., Park K., Rhee K. (2013). Heat stress response of male germ cells. *Cellular and Molecular Life Sciences*.

[5] Barazani Y., Katz B. F., Nagler H. M., Stember D. S. (2014). Lifestyle, environment, and male reproductive health. *Urologic Clinics of North America*.

[6] Prieto C., Suárez P., Bautista J. M. (1996). Semen changes in boars after experimental infection with porcine reproductive and respiratory syndrome (PRRS) virus. *Theriogenology*.

[7] Bansal A. K., Bilaspuri G. S. (2011). Impacts of oxidative stress and antioxidants on semen functions. *Veterinary Medicine International*.

[8] Zini A., Garrels K., Phang D. (2000). Antioxidant activity in the semen of fertile and infertile men. *Urology*.

[9] Fraczek M., Kurpisz M. (2007). Inflammatory mediators exert toxic effects of oxidative stress on human spermatozoa. *Journal of Andrology*.

[10] Depuydt C. E., Bosmans E., Zalata A., Schoonjans F., Comhaire F. H. (1996). The relation between reactive oxygen species and cytokines in andrological patients with or without male accessory gland infection. *Journal of Andrology*.

[11] Tremellen K. (2008). Oxidative stress and male infertility—a clinical perspective. *Human Reproduction Update*.

[12] Superchi P., Talarico L., Beretti V., Bonomi A. (2005). Effect of dietary administration of oil extract from rosemary on reproductive efficiency in boars. *Italian Journal of Animal Science*.

[13] Liu Q., Zhou Y., Duan R., Wei H., Jiang S., Peng J. (2016). Lower dietary n-6 : n-3 ratio and high-dose vitamin E supplementation improve sperm morphology and oxidative stress in boars. *Reproduction, Fertility and Development*.

[14] Yun S. J., Bae G.-S., Park J. H. (2016). Antioxidant effects of cultured wild ginseng root extracts on the male reproductive function of boars and guinea pigs. *Animal Reproduction Science*.

[15] Bouayed J., Rammal H., Dicko A., Younos C., Soulimani R. (2007). Chlorogenic acid, a polyphenol from *Prunus domestica* (Mirabelle), with coupled anxiolytic and antioxidant effects. *Journal of the Neurological Sciences*.

[16] Ghosh M. K., Chattopadhyay D. J., Chatterjee I. B. (1996). Vitamin C prevents oxidative damage. *Free Radical Research*.

[17] Zhang T., Zhou Y. F., Zou Y. (2015). Effects of dietary oregano essential oil supplementation on the stress response, antioxidative capacity, and HSPs mRNA expression of transported pigs. *Livestock Science*.

[18] Yin J., Liu M., Ren W. (2015). Effects of dietary supplementation with glutamate and aspartate on diquat-induced oxidative stress in pigletse. *PLoS ONE*.

[19] Kondoh T., Torii K. (2008). MSG intake suppresses weight gain, fat deposition, and plasma leptin levels in male Sprague-Dawley rats. *Physiology and Behavior*.

[20] Iwatsuki K., Torii K. (2012). Peripheral chemosensing system for tastants and nutrients. *Current Opinion in Endocrinology, Diabetes and Obesity*.

[21] Kong X. F., Zhou X. L., Feng Z. M. (2015). Dietary supplementation with monosodium l-glutamate modifies lipid composition and gene expression related to lipid metabolism in growing pigs fed a normal- or high-fat diet. *Livestock Science*.

[22] Zhang J., Yin Y., Shu X. G. (2013). Oral administration of MSG increases expression of glutamate receptors and transporters in the gastrointestinal tract of young piglets. *Amino Acids*.

[23] Kang P., Liu Y., Zhu H. (2014). The effect of aspartate on the energy metabolism in the liver of weanling pigs challenged with lipopolysaccharide. *European Journal of Nutrition*.

[24] Pi D., Liu Y., Shi H. (2014). Dietary supplementation of aspartate enhances intestinal integrity and energy status in weanling piglets after lipopolysaccharide challenge. *Journal of Nutritional Biochemistry*.

[25] Russell R. R., Taegtmeyer H. (1991). Changes in citric acid cycle flux and anaplerosis antedate the functional decline in isolated rat hearts utilizing acetoacetate. *The Journal of Clinical Investigation*.

[26] Duan J., Yin J., Ren W. (2016). Dietary supplementation with l-glutamate and l-aspartate alleviates oxidative stress in weaned piglets challenged with hydrogen peroxide. *Amino Acids*.

[27] Li F., Li Y., Tan B. (2016). Alteration of inflammatory cytokines, energy metabolic regulators, and muscle fiber type in the skeletal muscle of postweaning piglets. *Journal of Animal Science*.

[28] Wang R., Xiao Y., Opriessnig T. (2013). Enhancing neutralizing antibody production by an interferon-inducing porcine reproductive and respiratory syndrome virus strain. *Vaccine*.

[29] Shen J., Chen Y., Wang Z. (2014). Coated zinc oxide improves intestinal immunity function and regulates microbiota composition in weaned piglets. *British Journal of Nutrition*.

[30] Geng M., Li T., Kong X. (2011). Reduced expression of intestinal N-acetylglutamate synthase in suckling piglets: a novel molecular mechanism for arginine as a nutritionally essential amino acid for neonates. *Amino Acids*.

[31] Yin F., Zhang Z., Huang J., Yin Y. (2010). Digestion rate of dietary starch affects systemic circulation of amino acids in weaned pigs. *British Journal of Nutrition*.

[32] Wu L., Wang W., Yao K. (2013). Effects of dietary arginine and glutamine on alleviating the impairment induced by deoxynivalenol stress and immune relevant cytokines in growing pigs. *PLoS ONE*.

[33] Wang J., Cao Y., Wang C., Sun B. (2011). Wheat bran xylooligosaccharides improve blood lipid metabolism and antioxidant status in rats fed a high-fat diet. *Carbohydrate Polymers*.

[34] Chen J., Shen S., Tan Y. (2015). The correlation of aromatase activity and obesity in women with or without polycystic ovary syndrome. *Journal of Ovarian Research*.

[35] Zuo J., Ling B., Long L. (2015). Effect of dietary supplementation with protease on growth performance, nutrient digestibility, intestinal morphology, digestive enzymes and gene expression of weaned piglets. *Animal Nutrition*.

[36] Xiong X., Yang H. S., Wang X. C. (2015). Effect of low dosage of chito-oligosaccharide supplementation on intestinal morphology, immune response, antioxidant capacity, and barrier function in weaned piglets. *Journal of Animal Science*.

[37] Wang J., Li G. R., Tan B. E. (2015). Oral administration of putrescine and proline during the suckling period improves epithelial restitution after early weaning in piglets. *Journal of Animal Science*.

[38] Cutler R. G., Plummer J., Chowdhury K., Heward C. (2005). Oxidative stress profiling: part II. Theory, technology, and practice. *Annals of the New York Academy of Sciences*.

[39] Lobo V., Patil A., Phatak A., Chandra N. (2010). Free radicals, antioxidants and functional foods: impact on human health. *Pharmacognosy Reviews*.

[40] Agarwal A., Makker K., Sharma R. (2008). Review article: clinical relevance of oxidative stress in male factor infertility: an update. *American Journal of Reproductive Immunology*.

[41] Silver R., Kriegsfeld L. J. (2001). Hormones and behaviour. *eLS*.

[42] MacLean C. R. K., Walton K. G., Wenneberg S. R. (1997). Effects of the transcendental meditation program on adaptive mechanisms: changes in hormone levels and responses to stress after 4 months of practice. *Psychoneuroendocrinology*.

[43] Steiner E. T. (2011). Testosterone and vasopressin in men's reproductive behavior. *Department of Psychology*.

[44] Pitteloud N., Mootha V. K., Dwyer A. A. (2005). Relationship between testosterone levels, insulin sensitivity, and mitochondrial function in men. *Diabetes Care*.

[45] Turner T. T., Lysiak J. J. (2008). Oxidative stress: a common factor in testicular dysfunction. *Journal of Andrology*.

[46] Hess R. A. (2003). Estrogen in the adult male reproductive tract: a review. *Reproductive Biology and Endocrinology*.

[47] Wells R., Kenny A. L., Duckett R., Wreford N. G., Johnston S. D., D'Occhio M. J. (2013). Elucidation of the role of LH and FSH during neonatal testicular development and growth in the boar. *Animal Reproduction Science*.

[48] Kowluru R. A., Engerman R. L., Case G. L., Kern T. S. (2001). Retinal glutamate in diabetes and effect of antioxidants. *Neurochemistry International*.

[49] Reuter S., Gupta S. C., Chaturvedi M. M., Aggarwal B. B. (2010). Oxidative stress, inflammation, and cancer: how are they linked?. *Free Radical Biology and Medicine*.

[50] Gürbüz B., Yalti S., Fiçicioğlu C., Zehir K. (2003). Relationship between semen quality and seminal plasma total carnitine in infertile men. *Journal of Obstetrics and Gynaecology*.

[51] Zini A., San Gabriel M., Baazeem A. (2009). Antioxidants and sperm DNA damage: a clinical perspective. *Journal of Assisted Reproduction and Genetics*.

[52] Stoll B., Burrin D. G., Henry J., Yu H., Jahoor F., Reeds P. J. (1999). Substrate oxidation by the portal drained viscera of fed piglets. *American Journal of Physiology—Endocrinology and Metabolism*.

[53] Burrin D. G., Stoll B. (2009). Metabolic fate and function of dietary glutamate in the gut. *The American Journal of Clinical Nutrition*.

[54] Kocic G., Nikolic J., Jevtovic-Stoimenov T. (2012). L-arginine intake effect on adenine nucleotide metabolism in rat parenchymal and reproductive tissues. *The Scientific World Journal*.

[55] Rocio G. C., Saul C. R., Ines G.-C. S. (2013). Testicular expression of the TGF-*β*1 system and the control of Leydig cell proliferation. *Advances in Bioscience and Biotechnology*.

[56] Foey A. D., Parry S. L., Williams L. M., Feldmann M., Foxwell B. M. J., Brennan F. M. (1998). Regulation of monocyte IL-10 synthesis by endogenous IL-1 and TNF-*α*: role of the p38 and p42/44 mitogen-activated protein kinases. *Journal of Immunology*.

[57] Gunnett C. A., Heistad D. D., Berg D. J., Faraci F. M. (2000). IL-10 deficiency increases superoxide and endothelial dysfunction during inflammation. *American Journal of Physiology—Heart and Circulatory Physiology*.

